# Knowledge of partograph and its associated factors among obstetric care providers in North Shoa Zone, Central Ethiopia: a cross sectional study

**DOI:** 10.1186/s13104-015-1363-x

**Published:** 2015-09-04

**Authors:** Negash Wakgari, Gizachew Assefa Tessema, Abdella Amano

**Affiliations:** School of Nursing and Midwifery, College of Medicine and Health Sciences, Hawassa University, Hawassa, Ethiopia; Department of Reproductive Health, Institute of Public Health, University of Gondar, Gondar, Ethiopia; Department of Midwifery, College of Medicine and Health Sciences, University of Gondar, Gondar, Ethiopia

**Keywords:** Knowledge, Partograph, Obstetric care providers, North Shoa zone

## Abstract

**Background:**

Globally, there are 210 maternal deaths per 100,000 live births in 2013. Ethiopia is one of the ten countries contributing to 60 % of the global maternal deaths. Most of these deaths could be averted by enhancing safe motherhood strategies and providing skilled care at each delivery. This skilled care includes the use of partograph to monitor the progress of labor. With this aspect, this study is aimed to assess knowledge of partograph and its associated factors among obstetric care providers in North Shoa Zone, Central Ethiopia.

**Methods:**

An institution-based cross-sectional study was conducted in June, 2013. Four hundred three obstetric care providers were included in the study. A pre-tested and structured questionnaire was used to collect data. Data were entered into the Epi—Info software and exported to SPSS software for further analysis. Logistic regression analyses were used to identify the associated factors. Odds ratios with 95 % confidence interval (CI) were computed to determine the presence and strength of association.

**Results:**

In this study; 287 (71.2 %) of obstetric care providers had a good level of knowledge on the partograph. Working in the hospital [Adjusted odds ratio (AOR) = 2.71, P = 0. 027, 95 % CI 1.32, 5.57) and getting on the job training (AOR = 5.49, P = 0.001, 95 % CI 3.32, 9.08) were significantly associated with knowledge about partograph.

**Conclusions:**

A significant percentage of care providers had a good level of knowledge about partograph. Working in the hospital and getting on the job training were factors affecting provider’s knowledge on the partograph. The provision of on the job training is necessary to improve provider’s knowledge on the partograph. Moreover, giving a due attention for provider at health centers is also important.

**Electronic supplementary material:**

The online version of this article (doi:10.1186/s13104-015-1363-x) contains supplementary material, which is available to authorized users.

## Background

Globally, there were 210 maternal deaths per 100,000 live births in 2013. Sub-Saharan Africa has the highest regional maternal mortality ratio of 510 per 100,000 live births. Most of these deaths could be prevented with proven interventions, including improving the care provider’s knowledge and behavior in midwifery skills and ensuring women’s access to emergency obstetric care [1].

Ethiopia is one of the ten countries contributing to 60 percent of the global maternal deaths [2]. Despite child birth is universally celebrated event; a minimum of 20,000 Ethiopian women dies each year from pregnancy and childbirth related complications [3]. The maternal mortality ratio is 676 per 100,000 live births, which is the highest among the world [4]. The World Health Organization (WHO) recommends partograph utilization to help birth attendants to make an early diagnosis and management of prolonged and obstructed labor [5]. Despite partograph is simple and easily available; it is not widely used as it should be [6]. If correctly applied, it would help to reduce obstructed labor and its sequelae by alerting obstetric care providers to take an immediate action for any deviation from normal. Therefore, providers at all levels are expected to know how to use and interpret partograph correctly, and thus can reduce prolonged and obstructed labor and consequently maternal and perinatal mortality [7, 8].

Putting the partograph in the hands of the care provider and telling them to use it may not be sufficient. Rather, provision of on-job training is crucial for ensuring the obstetric care provider’s correct and up to date knowledge and skill about the tool [9]. Partograph is more helpful only in situations where obstetric care providers have adequate training in basic obstetric skills to be able to perform normal deliveries and vaginal examinations [10]. Different studies in developing countries depicted that the poor level of knowledge about partograph utilization among health care providers [11–13]. Hence, this study provides information on knowledge of partograph utilization and its associated factors in North Shoa Zone, Central Ethiopia.

## Methods

### Study setting

An institutional-based cross-sectional study was conducted in June, 2013 among obstetric care providers in North Shoa Zone, Central Ethiopia. North Shoa is one of the eleven zones in the Amhara National, Regional State. The zone includes 24 districts. Debre Birhan is the zonal capital, located at 130 km away from the Ethiopian capital, Addis Ababa. There are 4 government hospitals, 86 health centers, 387 health posts, one private hospital, 3 higher clinics, 7 medium clinics, and 93 lower clinics in the zone. Among privately owned facilities, one hospital and one higher clinic were the only delivery service providers. With regard to health professionals, there were about 354 clinical nurses, 121 midwives, 98 health officers, 45 BSc nurses, 29 general practitioners, and 1 obstetrician working in the zone during the study (Data obtained from each institution by principal investigator via preliminary survey).

### Study design

An institution-based cross-sectional study design was conducted to assess knowledge of partograph and its associated factors among obstetric care providers in North Shoa Zone, Central Ethiopia.

### Population

All obstetric care providers (midwives, nurses, general practitioners, and health officers working in both governmental and nongovernmental health institutions) in North Shoa Zone included in the study. Health professionals who did not attend labor cases in the Zone were excluded from participating in the study.

### Sample size and sampling procedure

The sample size was calculated using single population proportion formula: n = (Zα/2)^2^ × p (1 − p)/w^2^, considering the following assumptions: the proportion (p) for the level of knowledge on partograph 53.4 % based on previous study [13], 95 % confidence interval, 5 % of absolute precision, and 10 % of non response rate, the total sample size was calculated to be 421. Since the calculated sample size and study population was almost equal, we included all (403) eligible obstetric care providers. Thirty-one respondents were not available during the data collection period.

During the study period, 92 health institutions were providing delivery services in the Zone. The total numbers of health personnel who have been providing delivery services in these 92 institutions were 457 (Data obtained from each institution by principal investigator via preliminary survey).

### Data collection and tools

A pre-tested and structured self-administered questionnaire was used for data collection. Different relevant literatures were reviewed to develop the tool that addresses the objective of the study [9, 11–15]. The instrument was pretested on 30 study participants who were working in other health facilities which were not part of the actual study. Findings from the pre-test were used to modify the instrument in terms of clarifying the questions. The questionnaire was designed to obtain information on the socio-demographic and professional characteristics of care providers, knowledge of partograph, and their attitude towards partograph utilization. Knowledge about the partograph was measured by using eight knowledge questions. In order to produce a more objective assessment of knowledge about partograph a scoring method was devised and a knowledge score for each participant was obtained by summing up the score for correct responses given to the selected questions in the questionnaire. A score of mean value or above (≥4) to these questions were considered as a good level of knowledge, otherwise taken as poor level of knowledge [15] (Additional file 1).

Similarly, attitude towards partograph was measured using eight attitudes related questions. A 5-point Likert scale (with five responses = strongly agree, agree, undecided, disagree, strongly disagree) was used to measure providers’ attitude towards partograph utilization. An individual who responded strongly agree for positive attitude was given scores of 5 and 1 for those who responded as strongly disagree, while the above scores was reversed for negative attitude questions. Finally the total score (5 × 8 = 40) was dichotomized into favorable and an unfavorable attitude taking the mean score (20) as a cutoff point {Mean score or more (≥20) favorable attitude and less than the mean score (<20) unfavorable attitude}. A certified health personnel who provides care for women during labor and delivery were considered as an obstetric care provider. Two BSc nurses facilitated the data collection process. They were given one day training before the actual work about the aim of the study, procedures, and data collection techniques.

### Data analysis

The returned questionnaire was checked manually for their completeness and entered into Epi-Info version 3.5.1 statistical package, then exported to SPSS version 20.0 for further analysis. Data cleaning and cross-checking was done before analysis. Descriptive statistics were done. Both bivariate and multiple logistic regression analysis were used to determine the association of each independent variable with the dependent variable. Variables having p-value ≤0.2 in the bivariate analyses were entered into a multiple logistic regression model to control the confounding effect amongst the variables. Odds ratios with their 95 % confidence intervals were computed to identify the presence and strength of association, and statistical significance was declared if p < 0.05.

### Ethical considerations

Ethical approval was obtained from the Institutional Review Board of the University of Gondar. Permission letter was granted from the Zonal Health Department to respective health institutions. Written consent was obtained from each study subject prior to the data collection process. Participant’s involvement in the study was on a voluntary basis and participants who were unwilling to participate in the study and those who want to quit their participation at any stage were informed to do so without any restriction. To keep their confidentiality, personal identifiers were not utilized.

## Results

### Socio-demographic characteristics of study participants

A total of 403 obstetric care providers was included in the study (response rate = 95.7 %). About half 203 (50.4 %) of obstetric care providers were males. The mean age of the respondents was 26.2 years (SD = 4.26). Majority 324 (80.4 %) was working at the health center. Regarding their profession 183 (45.4 %) were nurses. Two hundred thirty-five (58.3 %) were diploma. The mean year duration of practice was 6.6 with extreme of 1 and 37 years (Table 1).

**Table 1:** Socio-demographic characteristics of obstetric care providers in North Shoa Zone, Central Ethiopia, 2013 (n = 403)

Variables	Frequency	Percentage
Sex		
Male	203	50.4
Female	200	49.6
Age in years		
20–24	184	45.7
25–29	180	44.7
30 or more	39	9.6
Health facilities they are working		
Health center	324	80.4
Governmental hospital	75	18.6
Private hospital	4	1
Profession		
Nurse	183	45.4
Midwifery	111	27.6
Health officer	88	21.8
General practitioner (MD)	21	5.2
Qualification level		
Diploma	235	58.3
Bsc	140	34.8
General practitioner	21	5.2
Msc	7	1.7
Year of experience (in years)		
1–5	237	58.8
6–10	140	34.8
≥11	26	6.4

### Knowledge about partograph utilization

Two hundred eighty-seven (71.2 %) of the care providers had a good level of knowledge of partograph utilization. More than half, 239 (59.3 %) were trained in partograph utilization.

The majority, 327 (81.1 %) knew that monitoring of labor using partograph is important to prevent obstructed labor and its sequelae. In addition, nearly three quarters 293 (72.7 %) knew that partograph is designed to detect deviation from normal delivery that would develop during labor progress. Two hundred fifty-six (63.5 %) of them knew that maternal blood pressure should be plotted on partograph at least every 4 h. Nearly two-thirds, 260 (64.5 %) and 253 (62.8 %) of the participants also identified partograph has a component in which maternal condition is recorded and the first partograph plots should fall on the alert line respectively.

With regard to cervical dilation, 264 (65.5 %) recognized that partograph plots should be started in the active phase of labor and 268 (66.5 %) of them mentioned it should be plotted on partograph every 4 h. Furthermore, 240 (59.6 %) knew that when cervical dilation moves to the right of the alert line it can indicate the presence of the slow progress of labor.

### Attitude of obstetric care providers towards partograph utilization

A significant percentage, 337 (83.6 %) of obstetric care providers in this study had a favorable attitude towards partograph. Nearly three quarters, 293 (72.7 %) of them agreed that partograph utilization can reduce maternal and newborn deaths. Nearly half, 195 (48.4 %) of the providers agreed that partograph is necessary to improve quality of care. A lower proportion, 138 (34.3 %) of care providers agreed that partograph is developed only for midwives. In contrast, more than half 239 (59.3 %) of obstetric care providers disagreed with the idea of partograph is a chart for monitoring labor by all obstetric care providers. More than half 223 (55.3 %) of the study participants agreed that partograph is a complex tool with a pictorial overview of labor, whereas 238 (59.1 %) of them agreed that partograph is a simple graphic recording of progress labor.

### Factors associated with knowledge about partograph utilization

In bivariate analysis the factors found to be significantly associated with knowledge about partograph utilization were: type of health facility, qualification, and year of experience and on the job training. However, health facility and getting on the job training on partograph were significantly associated with knowledge about partograph utilization in the multivariate logistic regression. Those obstetric care providers who were working in the hospital were 2.7 times more likely to have a good level of knowledge about partograph than those who were working in the health center (AOR = 2.71, P = 0. 027, 95 % CI 1.32, 5.57). In addition, those who received on job training were about 5.5 times more likely to have a good knowledge about partograph utilization than those who did not receive (AOR = 5. 49, P = 0. 001, 95 % CI 3.32, 9.08) (Table 2).

**Table 2:** Bivariate and multivariate analyses of factors associated with knowledge about partograph utilization among obstetric care providers, North Shoa, Central Ethiopia, 2013 (n = 403)

Variables	Knowledge about partograph		OR (95 % CI)		*P* value
	Good	Poor	COR (95 % CI)	AOR (95 % CI)	
Health facilities					
Hospitals	61	17	1.57 (1.14, 2.83)	2.71 (1.32, 5.57)	0.027
Health centers	226	99	1	1	
Qualification level					
Diploma	160	75	1		
BSc and above	127	41	1.45 (0.9, 2.19)		
Year of experience					
1–5	167	70	1		
6 and more	119	47	1.06 (0.68, 1.64)		
On-job training					
Yes	200	39	4.54 (2.86, 7.19)	5.49 (3.32, 9.08)	0.001
No	87	77	1	1	

## Discussion

This study assessed the level of knowledge about partograph utilization and associated factors among obstetric care providers in Shoa Zone, Central Ethiopia. In this study, (71.2 %) of obstetric care providers had a good level of knowledge about partograph utilization (95 % CI 67.0, 75.7). This finding is lower than the studies conducted in Addis Ababa (96.6 %) [14], Nigeria (84.2 %) [15], Gambia (80 %) [16]; however; it was higher than the studies conducted in Northwest Ethiopia (53.4 %) [13], Fawole AO, Nigeria (16.0 %) [12], (37.3 %) [11]. The discrepancies between our finding and other studies might be due to differences in data collection tools and differences in socio-demographic characteristics of study participants. For example, educational style of obstetric care providers in Nigeria may not be similar to that of Ethiopian obstetric care provider.

In the present study, the type of health facility where obstetric care providers working are significantly associated with knowledge of partograph utilization. Those who were working in the hospital were more knowledgeable about partograph than those who were working in the health center. This is consistent with the studies done in Nigeria [11] and Gambia [16]. This could be due to care providers working in the hospitals have an access to participate in bed side and morning session discussion with obstetricians than the care providers working in the health center. These opportunities would lead them to update their knowledge. The other possible explanation might be care providers working in tertiary level may have good perception towards partograph than those who are working in primary and secondary level of facilities [11].

The present study also showed that getting on the job training had a significant association with knowledge about partograph utilization. Obstetric care providers who trained on partograph were about 5.5 times more likely to have a good level of knowledge than their counterparts. A studies conducted in Northwest Ethiopia [13], Addis Ababa [14] and Nigeria supported this findings [11, 12]. This implies that unless they received refresher training on partograph; having formal education on partograph may not be adequate to have proper knowledge on it. WHO recommends that obstetric care providers should receive training in midwifery every 3–5 years [17]. In line with this, pre service and in service training on the partograph is important to improve the quality of care during labor by improving intrapartum care with partograph [18, 19].

As a cross-sectional study requires respondents to remember information retrospectively, recall and response bias are the potential limitations of this study. However, numerous scientific procedures have been employed to minimize the possible effects. To reduce the response bias, for instance, the detail about the aim of the study was shared with the respondents. In addition, procedures such as supervision, pretest of data collection tools, and adequate training of data collectors were utilized.

## Conclusions

A significant percentage of obstetric care providers in this study had a good level of knowledge about partograph utilization. The type of health facility they are working and the presence of on job training were significantly associated with knowledge of partograph. The provision of on-job training and giving special attention to providers working at the health center is important to improve knowledge about partograph.

## Additional file

Additional file 1. Study questionnaire to assess the knowledge of partograph.

## Abbreviations

AOR: adjusted odds ratio
CI: confidence interval
WHO: World Health Organization
