# Supplementary material for: Knowledge of partograph and its associated factors among obstetric care providers in North Shoa Zone, Central Ethiopia: a cross sectional study
Source: BMC Res Notes. 2015 Sep 4;8:407. doi: 10.1186/s13104-015-1363-x (PMC4558760; doi:10.1186/s13104-015-1363-x)
Supplement: Additional file 1. — Study questionnaire to assess the knowledge of partograph. [file 13104_2015_1363_MOESM1_ESM.docx]

**QUESTIONNAIRE**

**University of Gondar, College of medicine and health sciences,** **Department of Midwifery**

Questionnaires

The following questionnaire is aimed to assess obstetric care provider’s knowledge about partograph and associated factors among obstetric care providers, in North Shoa Zone, Central Ethiopia.

The purpose of this questionnaire is to gather information on provider’s knowledge on partograph and its associated factors among obstetric care providers.

I have identified you as a study participant hoping that you would be willing to help me by providing some information. I have several questions which I would like to ask you, if you have the time and are willing. The questionnaires include Socio-demographic characteristics, knowledge related questions, obstetric care providers and health facility related questions. All information you provide will be kept confidential. I will not include any identifiers, such as your name or exact address. Only honest answers would contribute to the improvement of health planning. Your role in the success of the research is important and I appreciate your contribution to the research. You can quit your participation at any time if you feel discomfort. There is no payment for your participation. It will take you some 25 minutes. Would this be okay with you?

I understood about the advantage of the research and the roles I will have in the research. I have agreed to participate in the research?

A. Yes

B. No

**Part-I Professional characteristics of obstetric care providers**

**Please, tick in the box below to indicate your response and write in the provided space for the following questions**

| Q101 | Your sex | Male 1  Female 2 |  |
| --- | --- | --- | --- |
| Q102 | How old are you? | _______years |  |
| Q103 | Which health facilities you are currently working? | Governmental hospital 1  Health center 2  Private hospital 3  Private clinics 4 |  |
| Q104 | Your profession? | Midwifery 1  Nurse 2  Health officer 3  General practitioner 4 |  |
| Q105 | Your qualification level? | Diploma 1  BSc 2  Msc 3  Medical Doctor 4 |  |
| Q106 | Year of experience (practice) | ______years |  |
| Q107 | Have you been received on-job training on partograph utilization? | Yes 1  No 2 |  |

**Part II: Knowledge related questions**

**Please tick one box for each statement below to indicate your response for the following questions**

| **Sr.No** | **Questions** | **Choices of answers** |  |
| --- | --- | --- | --- |
| Q301 | Monitoring labor by partograph is important to prevent obstructed labor. | Yes 1  No 2  Do not know 3 |  |
| Q302 | Partograph is designed to detect deviations from normal delivery that develop as labor progresses. | Yes 1  No 2  Do not know 3 |  |
| Q303 | Partograph has a component in which maternal condition will be recorded. | Yes 1  No 2  Do not know 3 |  |
| Q304 | The first partograph plots should fall on alert line. | Yes 1  No 2  Do not know 3 |  |
| 305 | Cervical dilation should be plotted on partograph every 4hrs. | Yes 1  No 2  Do not know 3 |  |
| Q306 | Cervical dilatation moves to the right of the alert line indicates slow progress of labor. | Yes 1  No 2  Do not know 3 |  |
| Q307 | Maternal blood pressure should be plotted on partograph at least every 4hrs. | Yes 1  No 2  Do not know 3 |  |
| Q308 | Partograph plots should be initiated in active phase, of labor (>4cm of cervical dilation). | Yes 1  No 2  Do not know 3 |  |
| **Part III: Attitude related questions**  **Please, tick one box to indicate to what extents do you agree or disagree with the following statement.** | | | |
| Q401 | Do you think that,partograph utilization can reduce maternal and newborn deaths | Strongly disagree 1  Disagree 2  Undecided 3  Agree 4  Strongly agree 5 | |
| Q402 | Do you think that, partograph can be used to diagnose prolonged labor. | Strongly disagree 1  Disagree 2  Undecided 3  Agree 4  Strongly agree 5 | |
| Q403 | Do you think that, partograph is necessary to improve quality of care? | Strongly disagree 1  Disagree 2  Undecided 3  Agree 4  Strongly agree 5 | |
| Q404 | Do you think that, partograph is a simple graphic recording of progress of labor? | Strongly disagree 1  Disagree 2  Undecided 3  Agree 4  Strongly agree 5 | |
| Q405 | Do you think that, partograph is relevant to prevent obstructed labor | Strongly disagree 1  Disagree 2  Undecided 3  Agree 4  Strongly agree 5 | |
| Q406 | Do you think that, partograph is a chart for monitoring labor by all obstetric care providers? | Strongly agree 1  Agree 2  Undecided 3  Disagree 4  Strongly disagree 5 | |
| Q407 | Do you think that, partograph is a complex tool with pictorial overview of labor? | Strongly agree 1  Agree 2  Undecided 3  Disagree 4  Strongly disagree 5 | |
| Q408 | Do you think that, partograph is developed only for midwives to monitor labor? | Strongly agree 1  Agree 2  Undecided 3  Disagree 4  Strongly disagree 5 | |

**Thank you for your time!**
